# Incarcerated Epigastric Hernia

**DOI:** 10.7759/cureus.22013

**Published:** 2022-02-08

**Authors:** Snehasis Das, Oseen Shaikh, Naveen Kumar Gaur, Gopal Balasubramanian

**Affiliations:** 1 Surgery, Jawaharlal Institute of Postgraduate Medical Education and Research, Puducherry, IND

**Keywords:** obstructed hernia, intestinal obstruction, meshplasty, incarcerated hernia, epigastric hernia

## Abstract

An epigastric hernia is a form of ventral hernia. Most of these contain preperitoneal fat or the omentum. Herniation of intra-abdominal organs, a rare form of rectus sheath midline herniations, is seldom seen, and incarcerations in these cases are rare. A 56-year-old male presented with complaints of irreducible epigastric swelling. Thorough clinical examination and imaging studies revealed that the patient had an epigastric hernia. An intraoperative image showed that the small bowel had herniated through the epigastric defect and was obstructed; however, the small bowel was viable. The contents were reduced after enlarging the constricting ring, and anatomical repair with mesh reinforcement was done. Postoperatively, the patient had an uneventful recovery and was discharged.

## Introduction

The most common forms of external hernias are inguinal, umbilical, femoral, and incisional [1]. Among these, epigastric hernias account for less than 4% of the cases [2]. The prolapse of the contents through a narrow defect is usually responsible for the constriction that threatens the viability of the contents. It starts from baseline incarceration to gangrene of the bowel. Early diagnosis and treatment are warranted. Surgical options are the treatment of choice. These can be anatomical repair, bowel resection stoma, anastomosis, or meshplasty, depending upon the patient's condition and bowel viability. An incarcerated epigastric hernia is a rare occurrence in the surgical literature and has been associated with significant eventuality. Herein, we report a rare case of incarcerated epigastric hernia in its early phase where the viability of the contents was restored through immediate exploratory surgery.

## Case presentation

**Figure 1:**
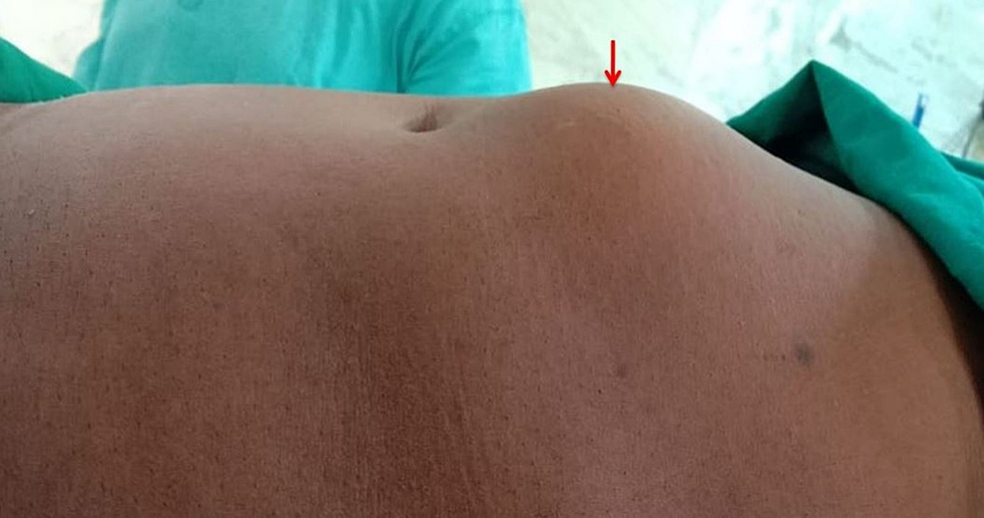
Clinical image showing irreducible epigastric hernia (arrow)

A 56-year-old male presented with complaints of a slow, progressive swelling in the upper abdomen for the last one month. He developed abdominal pain, vomiting, and obstipation for the last two days. The abdominal pain was colicky in type, in the epigastric region, moderate in intensity, with aggravation on food intake. The patient had five episodes of bilious vomiting per day, containing food particles, and was non-blood stained. On examination, the patient’s vitals were stable with tachycardia of 110 bpm and blood pressure of 110/70 mmHg. Abdominal examination showed a 1.5-cm epigastric defect, with the herniated small bowel forming a swelling of size 5 cm x 6 cm, irreducible, without any cough impulse (Figure 1).

**Figure 2:**
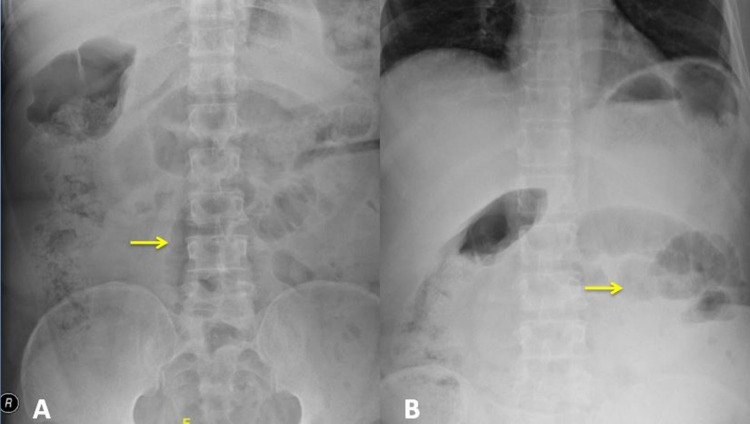
Abdominal X-ray showing (A) multiple dilated air small bowel loops (arrow) and (B) multiple air-fluid levels (arrow)

Blood tests showed normal hemoglobin, liver function, and renal function. Blood counts showed mild leukocytosis at 13,450 cells/mm^3^. The abdominal X-ray suggested dilated small bowel loops with features of intestinal obstruction (Figure 2).

**Figure 3:**
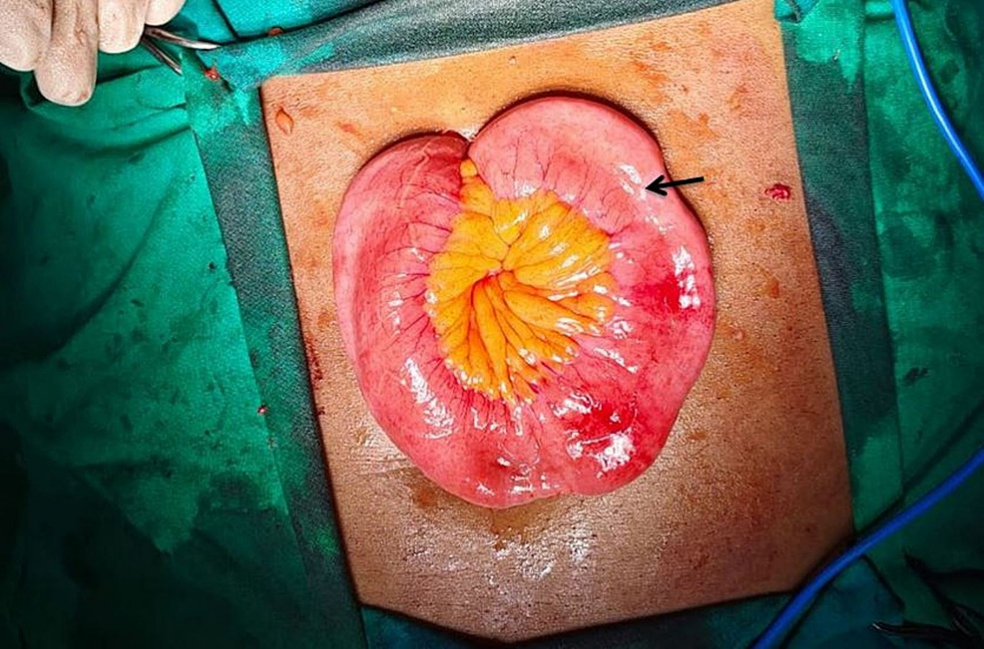
Intraoperative image showing herniated small bowel loop through the epigastric defect

The patient was diagnosed to have incarcerated epigastric hernia with intestinal obstruction. Hence, the patient was immediately taken for exploratory laparotomy. An intraoperative image showed extensive adhesions between the prolapsed contents and the edge of the defect in the abdominal wall (Figure 3).

The herniated loops were released from each other, and the constricting ring was released by extending the incision. The bowel was released from the hernia site and was viable, without any evidence of gangrenous changes. The contents were reduced, and primary closure of the hernia defect was done and reinforced with a non-absorbable mesh. Postoperatively, the patient improved without any complications.

## Discussion

The prolapse of tissue through a defect in the linea alba between the xiphoid process and the umbilicus is called an epigastric hernia. Anatomical involvement has seen predominance in the immediate 3 inches above the umbilicus while the incidence becomes rarer proximally. Epigastric hernias near the xiphoid process are rarely reported in the medical literature [3]. It has been seen that such hernias may stay asymptomatic for a long time, as long as 45 years, and reach massive sizes before getting incarcerated or strangulated [4]. It is most commonly seen in middle-aged men and obese patients, with risk factors ranging from coughing, heavy weight lifting, to chronic constipation and structural deformities.

These hernias are usually small in size, with preperitoneal fat as the usual content. The prolapse of intra-abdominal viscera is a rare occurrence, as the defect caused by the perforator vessels is usually very small. This is one of the oldest theories proposed by Moskowitz [5]. It has been reported in the literature that a true epigastric hernia with a sac is an infrequent clinical scenario, and most of the herniations had no sac, as in our case. In addition, a structural deformation theory by Aksar proposed the pattern of single aponeurotic decussation over a triple decussation, which is responsible for the weakness [6]. A true epigastric hernia with obstructed contents is a medical rarity and has been reported only four times so far in the medical literature [7,8].

Most of the patients remain asymptomatic for long periods, presenting with complaints of periodical pricking pain over the swelling, increasing food intake, and dyspepsia [5]. This may make the diagnosis of the obstructed hernia very difficult due to the unusual, atypical presentation. Clinicians should have a high level of suspicion while diagnosing an asymptomatic complicated epigastric hernia. Because of the small defect, the contents can degenerate into sudden irreducibility and then progress rapidly to obstruction. Diagnosis is usually confirmed through the clinical history, examination, and X-ray features. Our patient was diagnosed with an incarcerated epigastric hernia by clinical examination and X-ray features and hence immediately taken for laparotomy.

An incarcerated epigastric hernia is an emergency that most commonly requires immediate surgical exploration to relieve the obstruction. Although the progression from an incarcerated hernia to gangrene can be as fast as within six hours, early intervention can prevent gangrene of the contents. Healthy contents can be reduced into the abdomen, and a primary anatomical repair of the rectus sheath can be done. Depending on the preoperative state of the patient, secondary enforcement with mesh augmentation can be planned in the same setting or as a second surgery [9].

Moreover, in the case of gangrenous contents, urgent, rapid exploratory laparotomy is usually followed by resection of the gangrenous segment. Complicated epigastric hernias increase the risk of local complications, including surgical site infection, recurrence, and mesh infection, which can increase the morbidity of the patients [10,11]. In our case, as the contents were in the initial phase of incarceration, releasing the constriction resulted in complete resolution. We proceeded by reducing the contents with primary anatomical repair reinforced with a mesh.

## Conclusions

A complicated epigastric hernia is a rare phenomenon for which clinicians need to maintain a high risk of suspicion. Diagnosis is usually clinical, and imaging may be performed in doubtful cases. Due to the rapid deterioration, immediate diagnosis and prompt surgical intervention are the approaches of choice. This helps not only in avoiding unnecessary complications and the delay in recovery usually associated with resection and anastomosis of the gangrenous bowel segment but also in the expeditious recovery of the patient.
